# Percutaneous Lauromacrogol Foam Sclerotherapy for the Treatment of Acute Airway Compression Caused by Lymphatic Malformations in Infants

**DOI:** 10.1155/2018/3878960

**Published:** 2018-10-18

**Authors:** Lidan Wang, Fan Liu, Sui Huang

**Affiliations:** Department of Radiology, Wuhan Children Hospital, Tongji Medical College, Huazhong University of Science and Technology, Wuhan 430030, Hubei, China

## Abstract

Management of LMs still remains a challenge especially for those suffering from complications such as acute airway compression. In this study, we retrospectively evaluated the efficacy and safety of percutaneous lauromacrogol foam sclerotherapy for the patients with acute airway compression caused by lymphatic malformations (LMs) in infants. Five cases of infants with acute airway compression caused by LMs were treated with lauromacrogol foam sclerotherapy in the radiology department from February 2013 to August 2015 at Wuhan Medical and Healthcare Center for Women and Children, China. By CT examination and the DSA imaging, LMs were diagnosed and progressed cervical and sublingual LMs combined with hemorrhages were observed and suppression of the trachea was noticed as well, resulting in the difficulty with breathing and feeding. For all the patients, we extracted most cyst liquid from the LMs to reduce the surface tension and alleviate the respiratory pressure symptoms under the guidance of ultrasound. Subsequently, the lauromacrogol foam was injected percutaneously into the cyst of LMs. The dose of the agent was determined according to the size of the LMs, which was 3-8 ml in our study. After treatment, autonomous respiration and independent eating were observed. When the procedures were completed after 16 cycles, the cyst cavity became atrophic and then nearly vanished. During the follow-up period (a minimum of three months and a maximum of two years), 4 patients were clinically proved to be cured and one patient was significantly improved. There was no recurrence, serious complications, or adverse reactions. Our study demonstrated that percutaneous sclerotherapy combined with lauromacrogol foam is a safe, effective therapy for acute airway compression caused by LMs, especially giving a good cosmetic result.

## 1. Introduction

Lymphatic malformations (LMs) are considered to be a benign vascular lesion that arises from embryological disturbances in lymphatic system development. It can be found in any anatomic region and is more obvious in lymphatic-rich areas such as the head and neck (45–52%), axilla, mediastinum, groin, and retroperitoneum [1]. There are three morphologic types of cystic lymphatic lesions: macrocystic, microcystic, and the hybrid. Normally, the treatment methods clinically mainly include surgical resection, hardening, and laser treatment. According to disease type and scope of lesions, one way or combination of several methods can be considered. However, it is a significant challenge to treat LM due to the particular anatomical involvement between the lesion and the tongue, deep cervical space, and mediastinum [2]. Notably, the LM located at the neck is still challenging for the physician due to its complicated anatomical relationship among the surrounding vital tissues such as airway and nerves. Additionally, some fatal complication such as airway obstructions and impairment of oral feeding remains another obstacle for the choice of the treatment such as the surgery [3, 4]. Thus, a novel strategy by using a simple and safe procedure to preserve the normal anatomical structure around the lesion is worthy of evaluation.

Currently, it has been suggested that the sclerotherapy may serve as a promising and effective strategy to treat and resolve the LM especially for the macrocystic one [5]. Normally, sclerotherapy is performed by entering the cystic cavity by a direct puncture, aspirating the cystic fluid, and finally injecting the sclerosant [5]. It is believed that sclerotherapy may help to create the increased surface area between the sclerosant and the cyst wall, thus introducing endothelial inflammation in a vascular structure and giving rise to the thrombosis, fibrosis, and contraction toward the lesion. Featured by minimal invasion, simple operation, and low price, sclerotherapy has been widely applied clinically in recent years [6, 7]. Herein, in this study, we reported five cases of LMs treated by percutaneous sclerotherapy combined with lauromacrogol foam with good outcome, thus providing more options for this rare and complicated disease.

## 2. Methods

**Figure 1:**
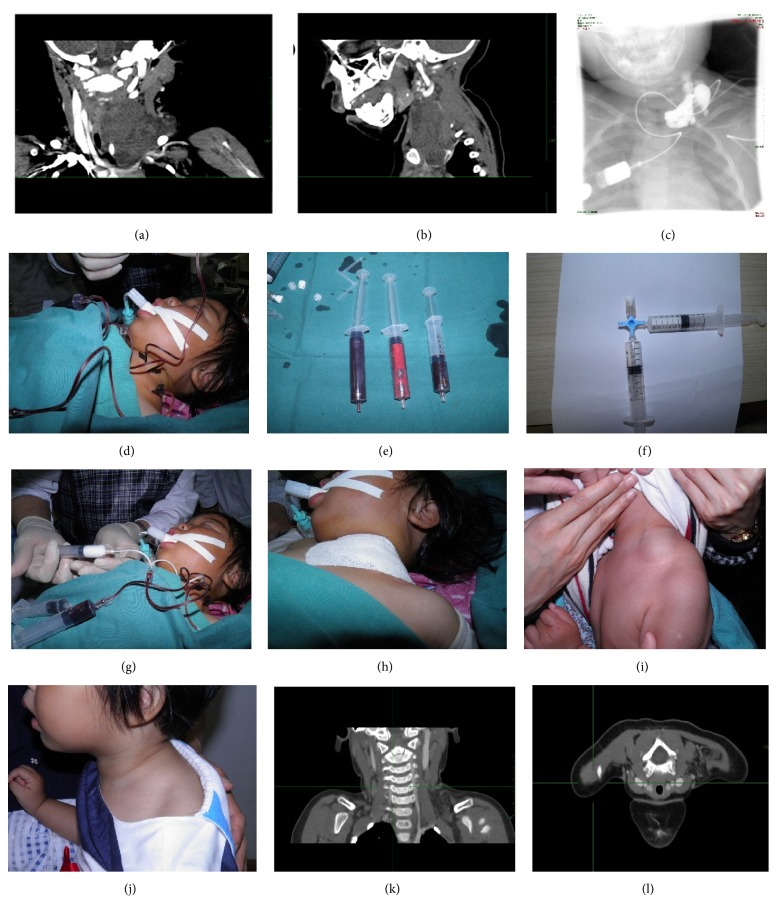
17-month-old female with lymphatic malformations (LMs) in the left cervical region that suddenly expanded over a 10-day period. The heart rate decreased and she underwent endotracheal intubation for dyspnea. (a)-(b) Computed tomography (CT) image revealing multilocular LMs (58 × 37 × 50 mm). The airway is obviously compressed and shifted. (c) Imaging for positioning. (d)-(e) Multiple points were percutaneously punctured and 25 mL of fluid was aspirated. (f) Preparation for the lauromacrogol foam injection. (g) Injection of the lauromacrogol foam. (h) The local compression bandage applied after treatment. (i) At the 1-month follow-up appointment, the mass was obviously shrunken. Three additional treatment sessions were then completed. (j) After 6 months, the mass disappeared. (k)-(l) The CT scan showing that the cyst was obviously decreased and the residual cystic cavity showed no lymph left.

Five patients (one male and four females with age ranging from 1 day to 17 months) were collected from February 2013 to August 2015.  Figure 1 shows a case of a 17-month-old girl. This study was approved ethically by Medical Ethics Committee of Wuhan University (NO. 2013008), and written informed consent was obtained from each participant. All cases were diagnosed as macrocystic LMs by computed tomography (CT) to identify the locations and their correlations to adjacent tissues: one was a single cyst, while the others were multilocular (Table 1). There is more than one anatomic zone affected in two cases: one in the cervical, retropharyngeal, parapharyngeal, and sublingual zones; the other one found in the cervical and superior mediastinum zones. Among them, the tongue was involved in one case. Four patients suffered from difficulty with breathing as the airway was suppressed by the accelerated enlargement of LM, of which three patients showed unstable vital signs, dyspnea, and the decreased heart rate that required cardiopulmonary resuscitation and endotracheal intubation respirator-assisted ventilation.

The sclerosants foams were premanufactured by using lauromacrogol liquid. Briefly, a three-way valve connecting two injection syringes was utilized: one contained 1% lauromacrogol liquid and the other contained air. Then, the syringes were injected toward each other approximately 20 times using the eddy method (Tessari) to create a liquid with the air proportion as 1:4.

Briefly, digital subtraction angiography (DSA) was used in advance to observe the sclerosants foam distribution and make sure that no venolymphatic malformations were determined. Patients received local anesthesia (n =1) and intravenous anesthesia (n = 4), respectively. Under the guidance of ultrasound, a 20-gauge needle was inserted in the LMs cyst cavity. All cyst liquid from the LMs was extracted to reduce the surface tension and the respiratory pressure. Subsequently, lauromacrogol foam was injected percutaneously into the cyst cavity. The dose of the agent was determined according to the size of the LMs, and the amount in this study was from 3 to 8 mL with average of 6.7 mL.

After the needle was removed, the inserted spot was pressured for 5–10 minutes to prevent exosmosis. The patients were discharged from hospital when the clinical symptoms improved after the first session. Next, a four-week follow-up was performed following each session. For those with the cyst, the patients should be treated in the outpatient department until the clinical symptoms disappeared or cyst was blocked.

Four cases of our group took percutaneous local puncture for emergently extracting 25-200 mL of hemorrhagic noncondensable fluid at the bedside. In one case of sublingual LM, 22 mL of a light yellow clear liquid was extracted. The symptoms of dyspnea and airway oppression were improved significantly after the first session of treatment. Thus, the respirator-assisted ventilation was ceased in one patient after the first session. In another two cases, the tracheal cannula was removed and the assisted ventilation was ceased after the second round of sclerotherapy treatment. In one case with a sublingual LM, two sessions of sclerotherapy treatment enabled the patients to eat independently prior to hospital discharge. The five patients were treated for a total of 16 sessions. The cyst cavity narrowed until they almost disappeared. The patients were followed up from 3 months to 2 years. Four cases were considered clinically cured, while the other one was considered significantly improved. However, the patient with improved outcome has been followed up for only three months; therefore we are confident that there will be good outcome with her. No recurrence or serious complication was observed. There were only two cases of local swelling after treatment, which were improved after localized puncture decompression. One case of local swelling was improved spontaneously after 24 hours without treatment. Hence, we concluded that local swelling was a common symptom after sclerotherapy treatment, which only required symptomatic treatment (Table 1).

## 3. Discussion 

Lymphatic malformations (LMs), also known as lymphangioma, have been thought to be caused by the congenital malformations that occurred at lymphatic vessel or the lymphatic fluid, thus causing retention, lymphangiectasia, and hyperplasia. Intralymphatic hemorrhage, infection, or trauma may lead to acute LM expansion and airway obstruction occasionally when the deep neck space is involved [8]. So far, the tracheotomy is inevitable for severe airway compromise. Berg et al. [3] previously proposed a staging system to help guide treatment and predict clinical outcomes for chronic obstruction of the pediatric airway with massive cervicofacial lesions. The authors claimed that decannulation was available for the patients at early stage compared to the patients with more severe condition. On the contrary, three patients in our study who underwent endotracheal intubation exhibited good breath spontaneously after treatment.

With respect to the clinical treatment against lymphatic malformations, surgical excision has been considered as the most effective way; however, complete resection is usually impossible, resulting in the high recurrence rate. Gilony et al. [9] reported that only 67% of the patients obtained complete resolution after surgery, 20% showed incomplete removal with fair cosmetic result, and 13% had a poor cosmetic result; however, 95% of lymphatic malformation patients showed an excellent response for sclerotherapy.

Injection sclerotherapy is a technique for introducing endothelial inflammation in a vascular structure by injecting a material, giving rise to the thrombosis, occlusion, fibrosis, and contraction. Sclerosant normally consists of picibanil (OK-432), doxycycline, bleomycin, ethanol, sodium tetradecyl sulfate, acetic acid, sotradecol, polidocanol, or lauromacrogol, etc. Previously, it has been applied successfully for the cyst located at the liver [6], variceal bleeding [7], etc. Among these multiple sclerosing agents, each agent has certain curative effect but there are also some limitations and side effects. For instance, bleomycin can cause pulmonary fibrosis or death, and OK-432 has the risk of an adverse reaction in patients with a penicillin G allergy and difficulties in acquiring the substance [10]. Lauromacrogol is an anionic surfactant consisting of polyoxyethylene lauryl ether, ethanol, and sterilized water [11]. Its effect is on the lipid molecules in the endothelial cells, inducing intimal inflammation and thrombosis formation, followed by the formation of fibrosis tissue and obliteration of the targeted vein [12]. Another potential advantage of lauromacrogol is a substantial reduction in pain because of its local anesthetic effect. Notably, in our study, we reported that sclerotherapy using lauromacrogol foam against LMs has shown obviously curative effects. Additionally, our findings clarified considerably fewer harmful reactions and less discomfort during the procedure, indicating that the sclerotherapy strategy using lauromacrogol foam is a safe and favorable therapy that can be applied in clinical activity.

In this study, our findings demonstrated that sclerotherapy therapy by using lauromacrogol foam on the head and neck of children with large LMs showed satisfied clinical outcome [13]. In addition, we utilized the method introduced by Wollmann's classic 1:4 liquid-gas ratio to manufacture the foam [14]. The foam showed excellent stability and enough tension for replacing cyst liquid. Therefore, the contact area and operating time with the cystic wall were increased, thus improving the clinical efficacy and safety with much less doses. We believe that this therapy is beneficial especially for the treatment of LMs with airway compression symptoms.

LM-induced acute airway compression was common especially for patients with large single cysts or multiple cysts of different sizes. Intralymphatic hemorrhage, infection, or trauma may lead to acute expansion and oppress the surrounding tissue [15]. Hence, active and effective treatment is necessary. Patients should be treated in the intensive care unit (ICU) once the sign of acute airway compression was observed, and endotracheal intubation should be provided as the standard procedure to ensure vital sign stability. Impellizzeri et al. [16] reported excellent outcome with complete disappearance of the lesion in 87.5% of cases by using CT-guided ethanol sclerotherapy for cervical cystic lymphatic malformations in children. In comparison, for the severe patients with LMs, ultrasound was more convenient and easily available for the patients in intensive care unit (ICU) compared with CT-guided treatment.

Normally, this therapy should be carried out at the ICU bedside and under the guidance of ultrasound when the percutaneous puncture treatment is performed as the precise percutaneous access to the lesions is technically challenging. Therefore, prior to the treatment, the cystic size, quantity, and number as well as the correlation between the adjacent organs and critical neurovascular structures need to be determined. The primary goal is to relieve the airway pressure. Relatively large cysts should be punctured and drained as much as possible to reduce the pressure, followed by the injection of lauromacrogol foam full of the cysts, and the total amount should be less than 8 ml. Additionally, the examination of the aspirated fluid may help to confirm the pathological characteristics [17].

For those patients with large polycystic masses with high surface tension, multiple punctures from different angles as well as multiple sessions are always required. In our study, limited session of injection is enough for LMs on the sublingual and superior mediastinum (cases 2 and 3) with single cysts, whereas more repeated sessions need to be completed for those patients on the neck and pharynx (cases 1, 4, and5) with more cyst cavities. Compared with the treatment with open surgery [9], better cosmetic results can be acquired in our study. Thus, clinicians are encouraged to consider that surgical method is not only one solution for LMs. Given the more concern about the cosmetic result by the patient, lauromacrogol foam sclerotherapy is more worthy of evaluation.

## 4. Conclusion 

Management of LMs still remains a challenge for clinicians, especially for those suffering from complications such as acute airway compression. Controlling the associated critical symptoms, maintaining functionality, and preserving aesthetic integrity are thought to be the ultimate goals. Compared with open surgery, lauromacrogol foam sclerotherapy should be considered as a safe and effective nonsurgical therapy for the infant patients with acute airway compression caused by LMs because of its less trauma and excellent outcome.

## Figures and Tables

**Table 1:** Clinical data.

Case	Sex	Age	Position	Size (cm)	Type	Endotracheal intubation	Decannulated	Sessions	Outcome	Follow-up	Complication	Imaging technique
1	F	1m	cervical, beside the pharynx, pharynx posterior, sublingual	6*∗*7*∗*9	macrocystic	no	-	5	cure	24 months	no	CT, ultrasound
2	M	1d	sublingual	3*∗*4*∗*5	macrocystic	no	-	2	cure	18 months	no	CT, ultrasound
3	F	6m	cervical, superior mediastinum	4*∗*5*∗*8	macrocystic	yes	2 days	2	cure	24 months	no	CT, ultrasound
4	F	17m	left cervical	4*∗*5*∗*6	macrocystic	yes	3 days	4	cure	6 months	local swelling	CT, ultrasound
5	F	12m	right cervical	6*∗*6*∗*10	macrocystic	yes	8 days	3	improved	3 months	local swelling	CT, ultrasound

## Data Availability

Lidan Wang et al. This is an open access article distributed under the Creative Commons Attribution License, which permits unrestricted use, distribution, and reproduction in any medium, provided the original work is properly cited.
